# Evaluation of the Combined Application of AFP, AFP-L3%, and DCP for Hepatocellular Carcinoma Diagnosis: A Meta-analysis

**DOI:** 10.1155/2020/5087643

**Published:** 2020-09-17

**Authors:** Xueying Wang, Yangyu Zhang, Na Yang, Hua He, Xuerong Tao, Changgui Kou, Jing Jiang

**Affiliations:** ^1^Division of Clinical Research, First Hospital of Jilin University, Changchun, Jilin Province, China; ^2^Department of Epidemiology and Biostatistics, School of Public Health, Jilin University, Changchun, Jilin Province, China

## Abstract

The role of *α*-fetoprotein (AFP) in the surveillance and diagnosis of hepatocellular carcinoma (HCC) has been questioned in recent years due to its low sensitivity and specificity. In addition to AFP, several new serum biomarkers, such as lens culinaris agglutinin-reactive fraction of AFP (AFP-L3) and des-gamma-carboxy prothrombin (DCP), have also been identified as useful HCC serological markers. However, the exact diagnostic value of the combinations of these biomarkers for detecting HCC in patients with liver disease remains unclear. Thus, we performed the current meta-analysis to assess performance of AFP+AFP-L3%+DCP for diagnosing HCC. Studies were systematically searched in PubMed, Embase, the Cochrane Library, CNKI, and WanFang Data databases. After full-text evaluation, 13 studies from 11 articles focusing on the combination of the three serum biomarkers for HCC detection were enrolled. Random-effects models were used due to the presence of heterogeneity. The pooled sensitivity and specificity for AFP+AFP-L3%+DCP were 88% and 79%, respectively. The area under the summary receiver operating characteristic (sROC) curve was 0.91, and the diagnostic odds ratio (DOR) was 28.33 (95% CI 16.78-47.83). Subgroup analysis showed that the pooled sensitivity and specificity of AFP+AFP-L3%+DCP in the diagnosis of HCC versus cirrhosis patients were 0.81 and 0.82, respectively. In conclusion, the combination of AFP, AFP-L3%, and DCP may prove to be useful in the diagnosis and screening of HCC.

## 1. Introduction

With approximately 841,000 new cases and 782,000 deaths annually, hepatocellular carcinoma (HCC) is predicted to be the sixth most commonly diagnosed cancer and the fourth leading cause of cancer-related death worldwide [1]. Chronic infection with hepatitis B virus (HBV) or hepatitis C virus (HCV), aflatoxin-contaminated food, heavy alcohol intake, and metabolic liver disease are risk factors for HCC [2], while cirrhosis represents the major known risk factor for HCC, independently from the liver disease underlying etiology [3]. HCC patients are often clinically diagnosed in the advanced stages and miss the best time for early treatment, resulting in a 5-year survival rate of <16% [4–6]. In contrast, early-stage HCC patients can be effectively treated by percutaneous ablation or surgical resection, which greatly improves the prognosis, with a 5-year survival rate of approximately 70% [7]. Thus, screening high-risk populations and the early diagnosis of HCC are imperative [8]. Due to the low sensitivity of ultrasound [9] and the weak ability to distinguish HCC in the background of cirrhosis [10], serum biomarkers can effectively assist the detection of cancer [11]. The serological marker detection method is simple, fast, noninvasive, easy for patients to accept, and sensitive, so it is commonly considered as a noninvasive test to detect early-stage HCC [12].

Alpha-fetoprotein (AFP) has been used as a serological marker for HCC since the 1970s [13]. A value > 400 ng/ml is thought to indicate HCC [14]. However, the AFP levels of 40% of HCC patients are normal, and elevated levels of AFP are also reported in patients with viral hepatitis, cirrhosis, pregnancy, and the presence of other tumors, such as germ cell tumors and gastric cancer [15]. Considering its low sensitivity, using AFP alone in practice is problematic, and there is an urgent need to identify potential complementary biomarkers for the diagnosis of HCC.

Recently, the lens culinaris agglutinin-reactive fraction of AFP (AFP-L3) and des-gamma-carboxy prothrombin (DCP) have been proven to be HCC-specific biomarkers in many studies [16–21]. AFP-L3 can detect the development of HCC earlier than AFP and shows potential ability for diagnosing AFP-negative HCC [20]. Previous studies have also demonstrated that DCP has higher sensitivity and specificity than AFP [22–24]. Although some studies suggest that the combination of these biomarkers showed better performance than a single biomarker for the detection of early-stage HCC [25–27], the practical application of three serum markers in the screening or clinical diagnosis of HCC is still controversial. Therefore, we conducted a meta-analysis to further assess the diagnostic performance of AFP+AFP-L3%+DCP and provide useful information for the application of triple biomarkers in the screening and diagnosis of HCC.

## 2. Materials and Methods

The meta-analysis was registered in PROSPERO (an international prospective register of systematic reviews, https://www.crd.york.ac.uk/PROSPERO/): CRD 42020164014. The study complied with the Preferred Reporting Items for Systematic Reviews and Meta-analyses of Diagnostic Test Accuracy studies (PRISMA-DTA) guidelines [28].

### 2.1. Literature Search

A systematic literature search was carried out to retrieve studies published prior to 20 August 2019 in PubMed, Embase, Cochrane Library, CNKI, and WanFang Data databases. The following keywords were used: “lectin-bound AFP or AFP-L3” and “DCP or prothrombin or Des-gamma carboxy–prothrombin induced by the absence of vitamin K or antagonist-II or PIVKA-II” and “Alpha-fetoprotein or AFP” and “hepatocellular carcinoma or liver neoplasms or hepatocellular cancer or HCC or small hepatocellular carcinoma or SHCC”. Additionally, the references of the included articles and relevant published studies were manually searched.

### 2.2. Inclusion and Exclusion Criteria

The inclusion criteria were as follows: (i) contained combined sensitivity and specificity data on serum or plasma AFP and AFP-L3% and DCP assays, (ii) the diagnosis of HCC was proven by histological examination or made based on the appropriate imaging characteristics according to accepted guidelines, and (iii) the controls were non-HCC patients with liver disease. The exclusion criteria were as follows: (i) duplications, reviews, meta-analyses, conference abstracts, case reports, letters, or other incomplete reports; (ii) nondiagnostic research; (iii) incomplete information; and (iv) repeated samples.

### 2.3. Data Extraction and Quality Assessment

Two reviewers independently extracted data from each eligible article. The data included authors, country, publication year, number of patients, the method of assay, sensitivity, specificity, type of the control group, and cutoff points for the biomarkers. We applied the Quality Assessment of Diagnostic Accuracy Studies (QUADAS) [29, 30] tool to evaluate the quality of the selected studies. Each of the 14 items was rated as “yes,” “no,” or “unclear,” and evaluators assigned scores of “1” for “yes” and “0” for “no” or “unclear.” An article that attained a final score of 10 or more was considered a high-quality article.

### 2.4. Statistical Analysis

We assessed the pooled sensitivity, specificity, likelihood ratios (LRs), and diagnostic odds ratio (DOR) together with their 95% confidence intervals (CIs) to determine the diagnostic ability of the biomarkers in these studies. In addition, we generated a summary receiver operating characteristic (sROC) curve that is useful for summarizing the diagnostic accuracy of multiple reports. To further confirm the three biomarkers' capacity to discriminate between high-risk population (patients with cirrhosis under surveillance for the risk of HCC development) and patients with a tumor (patients with a definite diagnosis of HCC), we performed a subgroup analysis including only studies that used as control population patients with cirrhosis. As a potential source of heterogeneity, the threshold effects were evaluated by Spearman's correlation coefficient. Heterogeneity was investigated using the *I*^2^ statistic. When the *I*^2^ value > 50%, heterogeneity was considered significant and a random effects model was applied; otherwise, a fixed effects model was selected. We also conducted a metaregression analysis to explain the source of the observed heterogeneity. Potential publication bias was assessed with Deeks' funnel plot asymmetry test.

We used Meta-Disc statistical software (version 1.4; Cochrane Colloquium, Barcelona, Spain) and Stata (version 13.0; Stata Corporation, College Station, TX, USA) to conduct all the statistical analyses. All *P* values were 2-sided, and differences were considered statistically significant at *P* < 0.05.

## 3. Results

### 3.1. Literature Assessment

**Figure 1:**
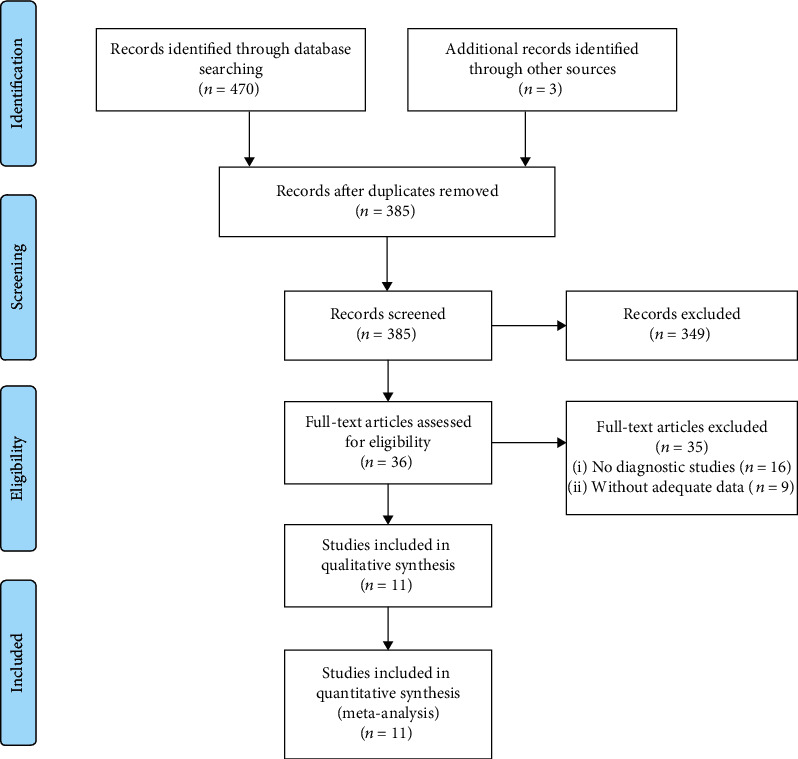
Flow diagram of the study selection process for eligible studies. ^∗^The searched databases and the number of articles are as follows: PubMed (119), Embase (206), Cochrane Library (15), CNKI (73), and WanFang Data (57).

The primary search of PubMed, Embase, the Cochrane Library, CNKI, and WanFang Data databases yielded 470 relevant articles. In addition, 3 articles were identified by manual searching. After 88 duplicates were excluded, the titles and abstracts of 385 articles were reviewed. Then, the full texts of 36 articles were retrieved; 16 articles did not describe HCC diagnostic tests, and 9 articles did not provide the proper data for constructing a 2 × 2 table. One of the articles contained three studies in different regions, and the population was not repeated, so all three studies were included in this meta-analysis. Ultimately, a total of 13 studies from 11 articles were enrolled in our study (Figure 1). We used the QUADAS tool to assess the quality of the papers; ten papers had a scores over 11, and one paper had a score of 10. Thus, all the included studies were of high quality according to the QUADAS assessment results. (See Table 1 in Supplementary Materials.)

The meta-analysis included 3516 HCC patients and 6081 controls. We extracted data from the selected articles, including the author, location, features of control, publication year, number of patients, test method, sensitivity, specificity, and cutoff points for the biomarkers (Table 1). The article of Berhane et al. [31] includes three cohorts from the UK, Japan, and Germany, and the diagnostic performance of the three biomarkers were showed in three cohorts individually, so Berhane et al. is listed three times in Table 1.

CLD: chronic liver disease; LiBASys: clinical autoanalyzer by a liquid-phase binding assay; IAUEC: immunometric assays utilizing enhanced chemiluminescence; ECLIA: immunoassay using the electrochemiluminescence detection system; EIA: conventional enzyme immunoassay; *μ*TAS assay: microchip capillary electrophoresis and liquid-phase binding assay; CMIA: chemiluminescent microparticle immunoassay.

### 3.2. Diagnostic Performance of AFP+AFP-L3%+DCP in HCC Detection

**Figure 2:**
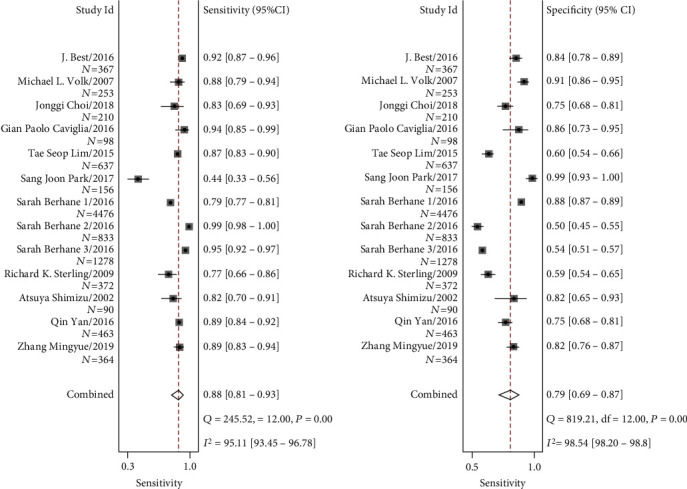
Forest plots of the sensitivity and specificity of AFP+AFP-L3%+DCP for the diagnosis of HCC. Sarah Berhane 1/2/3 are three studies in different regions from one article: (1) Japan; (2) the UK; (3) Germany.

**Figure 3:**
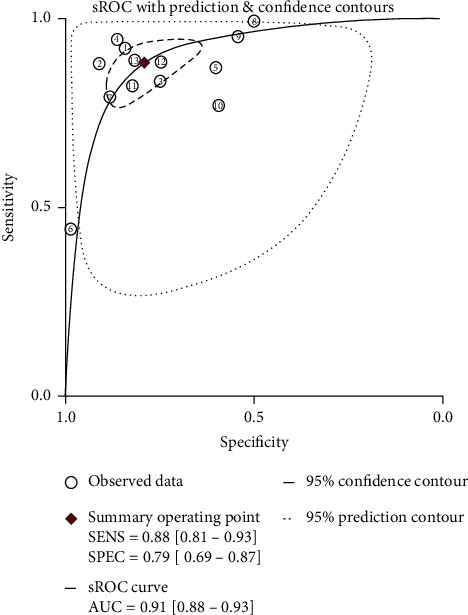
sROC curve of AFP+AFP-L3%+DCP for the diagnosis of HCC. ①: J. Best/2016; ②: Michael L.Volk/2007; ③: Jonggi Choi/2018; ④: Gian Caviglia/2016; ⑤: Tae Seop Lim/2015; ⑥: Sang Joon Park/2017; ⑦: Sarah Berhane/2016/Japan; ⑧: Sarah Berhane/2016/the UK; ⑨: Sarah Berhane/2016/Germany; ⑩: R. Sterling/2009; ⑪: Atsuya Shimizu/2002; ⑫: Qin Yan/2016; ⑬: Zhang Mingyue/2019.

A random effects model was applied due to the significant heterogeneity (*I*^2^ > 50%) in this meta-analysis.  Figure 2 shows that the pooled sensitivity and specificity of AFP+AFP-L3%+DCP in the diagnosis of HCC were 0.88 (95% CI 0.80-0.93) and 0.79 (95% CI 0.68-0.87), respectively. The pooled positive likelihood ratio (PLR) was 4.21 (95% CI 2.77-6.40), and the negative likelihood ratio (NLR) was 0.15 (95% CI 0.09-0.24). The diagnostic odds ratio (DOR) for the combination of the three markers was 28.33 (95% CI 16.78-47.83).  Figure 3 shows the summary receiver operator characteristic (sROC) curves of AFP+AFP-L3%+DCP, and the area under the curve (AUC) value was 0.91 (95% CI 0.88-0.93).

### 3.3. Heterogeneity Assessment and Metaregression

Heterogeneity was evaluated by the *I*^2^ test, and the *I*^2^ values for sensitivity, specificity, PLR, NLR, and DOR were 95.40%, 98.63%, 97.76%, 93.33%, and 100%, respectively. Considering the significant heterogeneity, we first assessed the threshold effects. The Spearman correlation coefficient was 0.412 (*P* = 0.162) for AFP+AFP-L3%+DCP, which suggested that there were no threshold effects in this meta-analysis. To further identify the source of heterogeneity, we conducted a metaregression to find potential confounders other than the cutoff effect (Table 2). The results of the metaregression demonstrated no significant heterogeneity with respect to the ethnicity (coefficient = −0.205, *P* = 0.6375), sample size (coefficient = 0.000, *P* = 0.3217), type of control group (coefficient = −1.217, *P* = 0.1473), or test method (coefficient = 0.140, *P* = 0.4416), which suggested that other factors might have caused the high heterogeneity in AFP+AFP-L3%+DCP.

### 3.4. Subgroup Analysis

**Figure 4:**
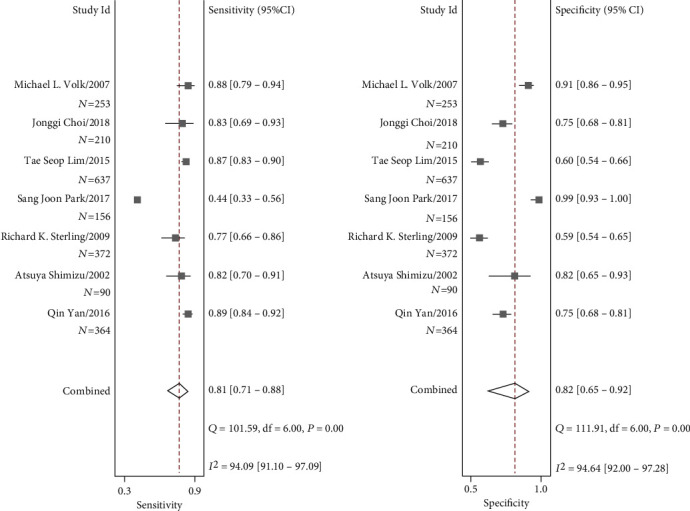
Forest plots of the sensitivity and specificity of AFP+AFP-L3%+DCP for the diagnosis of HCC versus cirrhosis patients.

We selected studies that only used cirrhosis patients as the control population to perform a subgroup analysis.  Figure 4 shows that the pooled sensitivity and specificity of AFP+AFP-L3%+DCP in the diagnosis of HCC discriminating cirrhosis patients were 0.81 (95% CI 0.71-0.88) and 0.82 (95% CI 0.65 -0.92), respectively. The pooled positive likelihood ratio (PLR) was 4.57 (95% CI 2.29-9.12), and the negative likelihood ratio (NLR) was 0.23 (95% CI 0.16-0.33). The diagnostic odds ratio (DOR) for the combination of the three markers was 19.66 (95% CI 9.94-38.86). And the area under the curve (AUC) value was 0.88 (95% CI 0.85-0.90).

### 3.5. Sensitivity Analysis and Assessment for Publication Bias

**Figure 5:**
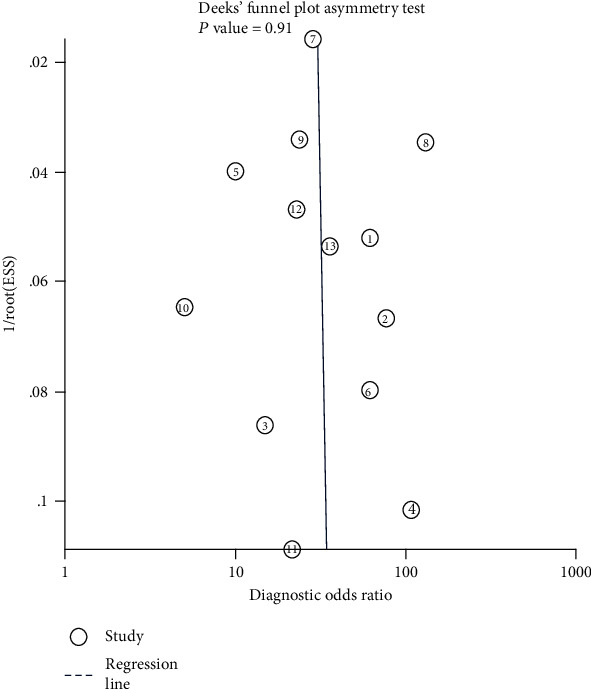
Deeks' funnel plot of AFP+AFP-L3%+DCP for the included studies. ①: J. Best/2016; ②: Michael L. Volk/2007; ③: Jonggi Choi/2018; ④: Gian Caviglia/2016; ⑤: Tae Seop Lim/2015; ⑥: Sang Joon Park/2017; ⑦: Sarah Berhane/2016/Japan; ⑧: Sarah Berhane/2016/the UK; ⑨: Sarah Berhane/2016/Germany; ⑩: R. Sterling/2009; ⑪: Atsuya Shimizu/2002; ⑫: Qin Yan/2016; ⑬: Zhang Mingyue/2019.

We removed individual included studies to evaluate the influence of the remaining data set on the sensitivity and specificity. The results of the sensitivity analysis demonstrated that the pooled results were stable, and there were no substantial alterations after removing studies. With respect to publication bias, Deeks' funnel plot asymmetry test (Figure 5) indicated no potential publication bias in the included studies, *P* = 0.91.

## 4. Discussion

The present meta-analysis demonstrated that the overall sensitivity and specificity of AFP+AFP-L3%+DCP were 88% and 79%, respectively. It is suggested that the combined application of three serum markers for HCC diagnosis shows a rather preferred performance. What is more, in the subgroup analysis including only studies that used cirrhosis patients as a control population, AFP+AFP-L3%+DCP also showed a good diagnostic performance, with the pooled sensitivity and specificity of 0.81 and 0.82, respectively. The results suggested that the combined application of AFP+AFP-L3%+DCP might be useful for the detection for the discovery of HCC patients and clinical decision-making.

At present, the most commonly used method for the early diagnosis of HCC is AFP combined with ultrasound. A meta-analysis conducted by Tzartzeva et al. [9] found that the sensitivity of ultrasound with AFP for detecting early-stage HCC was 63% (95% CI 48%-75%). Although the addition of AFP significantly increases sensitivity compared to ultrasound alone (45%, 95% CI 30%-62%), the result is still not satisfactory in practical screening or early diagnosis of HCC. In addition, ultrasound examination is highly dependent on the experience of the ultrasound doctor and the quality of equipment, so the results may not be as accurate and reliable as serological indicators.

For serum biomarkers, an AFP threshold of 400 ng/ml is typically used to diagnose HCC. However, the summary sensitivity of AFP alone is as low as 0.32 (95% CI 0.31-0.34) when the threshold is 400 ng/ml [39]. For AFP+DCP, a recent meta-analysis [40] found that the pooled sensitivity was 82%, and the pooled specificity was 85%. However, the results were based on the controls in the meta-analysis containing healthy people. In practice, AFP-L3 is currently tested at a percentage of AFP (AFP-L3%). With respect to AFP and AFP-L3%, Leerapun et al. [41] found a sensitivity of 71% and specificity of 63% with a total AFP ranging from 10 to 200 ng/ml and AFP‐L3% > 10% for HCC diagnosis. When AFP-L3% was greater than 35%, the sensitivity was reduced to 33%, while the specificity was increased to 100%. The results of a meta-analysis demonstrated that AFP-L3 alone (AUC = 0.710) was not inferior to the combination with AFP (AUC = 0.748) [42]. In our study, the combined application of the three biomarkers showed an overall better diagnostic performance, with a high sensitivity of 88% and a relatively satisfactory specificity of 79%. Moreover, the controls in our study were all CLD or cirrhosis patients, and thus, the results might be more useful for detecting HCC in patients with liver disease.

DOR is the ratio of PLR to NLR, and this ratio reflects the degree to which the results of a diagnostic test are related to the disease. When the value is greater than 1, a higher ratio indicates a better discriminatory diagnostic performance of the test. In our study, the pooled DOR value of AFP+AFP-L3%+DCP was 28.33, which indicates that the combination of triple biomarkers has a high accuracy for the diagnosis of HCC. The previous meta-analysis reported that the AUC for AFP+DCP was 0.90 [33], and that for AFP+AFP-L3 was 0.748 [36], while the results of our study showed that the AUC for AFP+AFP-L3%+DCP was 0.91. Our results indicated that the overall accuracy of the three combined markers is promising in the diagnosis and screening of HCC.

In this study, significant heterogeneity was present, but Spearman's correlation coefficient value revealed no threshold effects. A metaregression was conducted to identify the source of the heterogeneity. Potential confounders were inspected, including ethnicity, sample size, test method, and type of control. Nevertheless, the metaregression results demonstrated that none of these factors explained the heterogeneity in the performance of the three combined markers in HCC detection. The heterogeneity might be due to other factors such as differences in the clinical characteristics (liver disease etiology, tumor diameter) or the source of the patients (clinical patients or screening objects). We tried to obtain the above information from the original literature, but unfortunately, the data in the studies were insufficient or difficult to extract, which limits our exploration of heterogeneity.

The American Association for the Study of Liver Diseases (AASLD) and the European Association for the Study of the Liver (EASL) both recommend HCC surveillance using ultrasound alone. The Chinese Liver Cancer Diagnosis and Treatment Guidelines recommend ultrasound combined with serum AFP levels as monitoring methods. The Japanese guidelines recommend AFP-L3 and DCP as two additional serum tests for early-stage HCC detection [43]. In Japan, AFP, DCP, and AFP-L3 were covered by the national health insurance for HCC surveillance in 2002 [43]; in 2016, the detection rate of early-stage HCC patients and the 5-year survival rate were increased to 68% and 46.6% [31, 44], respectively. These rates are higher than those in China and the United States, indicating the promising value of triple biomarkers in early HCC diagnosis. However, no meta-analysis has been conducted to explore the value of the combination of the abovementioned biomarkers for HCC diagnosis. In this study, we evaluated the combined value of AFP+AFP-L3%+DCP for the diagnosis of HCC. All the included articles were of good quality according to the QUADAS assessment, ensuring the validity of the results. In addition, we also conducted a metaregression to explore the potential source of heterogeneity. Inevitably, limitations still existed in this research. Because of the limited previous studies on the combination of AFP, DCP, and AFP-L3%, only 13 studies from 11 articles were included in the present meta-analysis. In addition, due to the limited information of the included studies, such as the tumor size and etiology, we failed to identify the real source of significant heterogeneity. Moreover, the practical value of the combined biomarkers should not be measured by the high sensitivity and specificity only, and reasonable and acceptable cost-effectiveness should also be taken into consideration. One-stage mass abdominal ultrasound (AUS) screening has been shown to be more cost-effective than two-stage biomarker-AUS screening [45]. Thus, further studies are required to shed light on whether the improved effectiveness is worth the increased costs of additional biomarkers in the diagnosis and screening of HCC in a specific group of patients.

## 5. Conclusions

The results of our meta-analysis suggested that the combination of AFP+AFP-L3%+DCP showed an overall preferred diagnostic performance and may be helpful in the diagnosis and screening of HCC. In the future, research with a larger number of studies and sample sizes is required to further confirm the value of triple biomarkers in HCC diagnosis.

## Figures and Tables

**Table 1:** Characteristics of included studies.

Study	Year	Country	Control	Number	Test methods	Sensitivity	Specificity	Cutoff (AFP/AFP-L3%/DCP)
Best et al. [25]	2016	Germany	CLD	367	*μ*TAS assay	0.92	0.84	10 ng/ml, 10%, 7.5 ng/ml
Volk et al. [32]	2007	The US	Cirrhosis	253	IAUEC, EIA	0.88	0.91	23 ng/ml, 3%, 150 mAU/ml
Choi et al. [33]	2018	Korea	Cirrhosis	210	CMIA, *μ*TAS assay, ECLIA	0.83	0.75	5 ng/ml, 4%, 20 mAU/ml
Caviglia et al. [27]	2016	Italy	CLD	98	*μ*TAS assay	0.94	0.86	5.3 ng/ml, 1%, 0.4 ng/ml
Lim et al. [26]	2015	Korea	Cirrhosis	637	*μ*TAS assay, EIA	0.87	0.60	20 ng/ml, 5%, 40 mAU/ml
Park et al. [34]	2017	Korea	Cirrhosis	156	*μ*TAS assay	0.44	0.99	10 ng/ml, 7%, 40 mAU/ml
Berhane et al. [31]	2016	Japan	CLD	4476	*μ*TAS assay	0.79	0.88	20 ng/ml, 7%, 0.48 ng/ml
Berhane et al. [31]	2016	The UK	CLD	833	*μ*TAS assay	0.99	0.50	20 ng/ml, 7%, 0.48 ng/ml
Berhane et al. [31]	2016	Germany	CLD	1278	*μ*TAS assay	0.95	0.54	20 ng/ml, 7%, 0.48 ng/ml
Sterling et al. [35]	2009	North America	Cirrhosis	372	LiBASys	0.77	0.59	20 ng/ml, 10%, 7.5 ng/ml
Shimizu et al. [36]	2002	Japan	Cirrhosis	90	ECLIA	0.82	0.82	20 ng/ml, 10%, 40 mAU/ml
Qin et al. [37]	2016	China	Cirrhosis	463	*μ*TAS assay	0.89	0.75	20 ng/ml, 5%, 40 mAU/ml
Zhang et al. [38]	2019	China	CLD	364	LiBASys	0.89	0.82	7 ng/ml, 10%, 40 mAU/ml

**Table 2:** Metaregression analyses of the heterogeneity in AFP+AFP-L3%+DCP.

Variables	Coeff.	Std.Err.	*P* value	RDOR	(95%)CI
Ethnicity	-0.21	0.42	0.64	0.81	(0.31; 2.18)
Population	0.00	0.00	0.32	1.00	(1.00; 1.00)
Control	-1.22	0.75	0.15	0.30	(0.05; 1.73)
Test method	0.14	0.17	0.44	1.15	(0.77; 1.73)

^∗^Ethnicity was divided into Asians, Europeans, and North Americans.

## Data Availability

If the raw data of our study is needed, please contact our corresponding authors.

## Supplementary Materials

Supplementary Materials Table 1 contains the QUADAS assessment results of the included articles.
