# Supplementary material for: Evaluation of the Combined Application of AFP, AFP-L3%, and DCP for Hepatocellular Carcinoma Diagnosis: A Meta-analysis
Source: Biomed Res Int. 2020 Sep 17;2020:5087643. doi: 10.1155/2020/5087643 (PMC7519464; doi:10.1155/2020/5087643)
Supplement: Supplementary Materials — Table 1 contains the QUADAS assessment results of the included articles. [file 5087643.f1.doc]

**Supplementary Table** QUADAS assessment of included articles

| Author | Item1 | Item2 | Item3 | Item4 | Item5 | Item6 | Item7 | Item8 | Item9 | Item  10 | Item  11 | Item  12 | Item  13 | Item  14 | Q |
| --- | --- | --- | --- | --- | --- | --- | --- | --- | --- | --- | --- | --- | --- | --- | --- |
| J. Best. | Y | U | Y | U | Y | Y | Y | Y | Y | N | Y | Y | Y | Y | 11 |
| Michael L. | Y | U | Y | Y | Y | Y | U | Y | Y | N | Y | Y | Y | Y | 11 |
| Jonggi Choi | Y | U | Y | Y | Y | Y | Y | Y | Y | N | Y | Y | Y | Y | 12 |
| Gian Paolo Caviglia | Y | U | Y | Y | Y | Y | Y | Y | Y | N | Y | Y | Y | Y | 12 |
| Tae Seop  Lim | Y | Y | Y | U | Y | Y | Y | Y | Y | N | Y | Y | U | Y | 11 |
| Sang Joon Park | Y | Y | Y | U | Y | Y | Y | Y | Y | N | N | Y | Y | Y | 11 |
| Sarah Berhane | Y | Y | Y | Y | Y | Y | Y | Y | Y | N | Y | Y | Y | Y | 13 |
| Richard  K.Sterling | Y | Y | Y | Y | Y | Y | Y | Y | Y | N | N | Y | Y | Y | 12 |
| A. Shimizu | Y | Y | Y | Y | Y | Y | Y | Y | Y | N | N | Y | Y | Y | 12 |
| Qin Yan | Y | Y | Y | U | Y | Y | Y | Y | Y | N | N | Y | Y | Y | 11 |
| Zhang MY | Y | Y | Y | U | N | Y | Y | Y | U | N | Y | Y | Y | Y | 10 |

Item 1: Was the spectrum of patients representative of the patients? Item 2: Were selection criteria clearly described? Item 3: Isthe reference standard likely to classify the target condition? Item 4: Is the time period between reference standard and indextest short enough? Item 5: Did the whole sample use a reference standard of diagnosis? Item 6: Did patients receive the samereference standard regardless of the index test result? Item 7: Was the reference standard independent of the index test? Item8: Was the index test described in sufficient detail? Item 9: Was the reference standard described in sufficient detail? Item 10:Were the index test results interpreted without knowledge of the results of thereference standard? Item 11: Were the referencestandard results interpreted without knowledge of the results of the index test? Item 12: Were the same clinical data availablewhen test results were interpreted as would be available when the test is used in practice? Item 13: Were uninterpretable/intermediate test results reported? Item 14: Were withdrawals from the study explained?

Y: yes; N: no; U: unclear
